# Differential expression of a new isoform of DLG2 in renal oncocytoma

**DOI:** 10.1186/1471-2407-6-106

**Published:** 2006-04-26

**Authors:** Dmitry Zubakov, Zorica Stupar, Gyula Kovacs

**Affiliations:** 1 Laboratory of Molecular Oncology, Medical Faculty, Ruprecht-Karls-University, Heidelberg, Germany

## Abstract

**Background:**

Renal oncocytoma, a benign tumour of the kidney, may pose a differential diagnostic problem due to overlapping phenotype with chromophobe renal cell carcinoma or other types of renal cell tumours. Therefore, identification of molecular markers would be of great value for molecular diagnostics of this tumour type.

**Methods:**

In the current study we applied various techniques, including Affymetrix microarray hybridization and semiquantitative RT-PCR, to identify genes expressed differentially in renal oncocytomas. Subsequently, we used RACE and Northern blot hybridization to characterize the potential candidates for molecular diagnosis.

**Results:**

We have identified new isoform of DLG2 gene, which contains 3'-end exons of the known DLG2 gene along with the hypothetical gene FLJ37266. The new isoform is specifically upregulated in renal oncocytoma, whereas the known DLG2 gene is downregulated in this type of kidney tumour.

**Conclusion:**

The new isoform of DLG2 is the promising candidate gene for molecular differential diagnostics of renal oncocytoma.

## Background

Renal oncocytoma (RO) is a benign tumour of the kidney accounting for approximately 5% of renal cell tumours. A typical RO shows a nested or acinar growth of polygonal cells with central small nuclei and abundant eosinophilic granular cytoplasm when stained with H&E. However, RO as well as the malignant chromophobe renal cell carcinoma (chRCC) may display tubulary, cystic or papillary growth pattern and the cellular characteristics also may vary from small cells with scanty cytoplasma through large eosinophilic granular to "clear" pale cells even within the same tumour. Therefore, the differential diagnosis between the benign RO and some chRCC based exclusively on the cellular characteristics can be difficult. Positive staining with Hale's colloidal iron stain and the presence of cytoplasmic vesicles are considered to be diagnostic features for, but it is not limited to chRCCs [1-3]. An overlapping cytological and growth pattern may also be seen in other malignant renal cell tumours such as conventional RCC (cRCC) and papillary RCC (pRCC).

Previously, we have worked out a novel classification and differential diagnostic system based on the specific genetic changes occurring in conventional, chromophobe and papillary RCCs [4,5]. ROs are characterised either by loss of chromosomes 1 and 14 and the Y-chromosome or translocation between chromosome 11q13 and another chromosomes or by random genetic changes [6,7]. The lack of genetic changes specific for other types of renal cell tumours such as chRCC, pRCC and cRCC may indicate the diagnosis of RO [5,6,8]. Different molecular techniques such as microsatellite allelotyping and matrix CGH can be used to detect tumour type specific genetic alterations which require specific equipments and personal experienced in these techniques [5,9]. Several immunhistochemical markers such as CD63, MOC31, CD10, CDH16, KIT and RCC have been proposed to diagnose RO but none of them have been improved [10-13]. In course of a project of gene expression profiling in different types of renal tumours with Affymetrix microarray hybridization and semiquantitative RT-PCR we have identified a novel isoform of DLG2 gene upregulated exclusively in RO.

## Methods

### Tissue samples and RNA isolation

Tissue samples were collected from patients underwent tumour nephrectomy. A piece of tumour and corresponding normal kidney were snap-frozen immediately after nephrectomy in liquid nitrogen and stored at -80°C. The remaining tissue was fixed in 4% buffered formaldehyde for histological report. Histological diagnosis of tumours was established according to the Heidelberg Classification of Renal Cell Tumours [14]. Total RNA was prepared using modified method of Chomczynski & Sacchi [15]. The concentration and the integrity of RNAs were determined by spectrophotometry and denaturing agarose gel electrophoresis. The absence of DNA contamination in RNA samples was confirmed by PCR of GAPDH with primers specific to genomic DNA. The collection and use of all tissue samples for this study were approved by the Ethics Committee of the University of Heidelberg.

### Affymetrix GeneChip Array

We used the Affymetrix Human Genome U133 A and B GeneChip sets [16] to establish the gene expression in normal kidney tissues and distinct types of renal cell tumours. In this experiment we have pooled 3–5 RNAs each from normal healthy fetal and adult kidneys, Wilms' tumours, conventional, chromophobe and papillary RCCs and ROs. The cRNA synthesis, hybridisation onto Affymetrix Human Genome U133 GeneChip and primary statistical analysis were performed by the Affymetrix Screening Service at the German Resource Center for Genome Research (RZPD). The selection of genes expressed differentially in ROs (and other types of tumours) was performed using quantitative and qualitative measures such as signal log ratio, presence/absence and increase/decrease calls provided with Affymetrix Microarray Suit software (MAS.1). Genes showing a signal log ratio over 2 in ROs and under 0 in all other samples with corresponding detection and change call, i.e. specifically upregulated in ROs and down regulated in all other type of tissues were selected for quantitative RT-PCR.

### Semiquantitative RT-PCR

The results of microarray analysis were validated with RT-PCR on a panel of 40 cDNA samples representing 8 individual cases of each type of renal cancer, e.g. RO, chRCC, cRCC and pRCC as well as normal adult kidney. One microgram of total RNA was used for reverse transcription (Invitrogen, Superscript II kit), cDNA was denatured and diluted to 70 μl, 4 μl of this were used for PCR. The reactions were performed using Platinum Sybr Green qPCR UDG Supermix (Invitrogen) in a total volume of 15 μl. Detection of PCR product amplification was made by DNA Engine Opticon system (MJ Research). PCR specificity was controlled by melting curve analysis and agarose gel electrophoresis. Semi-quantification was performed with Opticon Monitor software (MJ Research). Expression of GAPDH and β-actin genes was used for the normalization of amplification signals in different samples. The evaluation of the results was done with statistical analysis tools of MS Excel.

### Rapid amplification of cDNA ends (RACE) and sequencing

SMART RACE cDNA amplification kit (BD Biosciences) was used to perform 3'- and 5'-RACE of FLJ37266 gene. RACE-ready cDNA was synthesized according to the manufacturer's instructions. RACE PCR was performed with gene-specific primers that were used for RT-PCR of FLJ37266 (Table 1, primers Affy_FLJ37266-F and -R). RACE products were excised form the agarose gel, purified, and cloned with TOPO TA Cloning kit (BD Biosciences). Clone inserts were sequenced with LiCor 4200 DNA sequencer using ThermoSequenase Primer Cycle kit (Amersham Biosciences) with IR700- and IR800-labelled primers (Table 1). Long distance (LD) exon-specific PCR was performed using the Advantage Polymerase mix (BD Biosciences). The list of primers used in the current work is presented in Table 1.

### Northern blot hybridization

Total RNA from normal and tumour tissues (10 μg) was size-fractionated in 1% denaturing agarose gel and transferred to membrane (N-Hybond, Amersham Biosciences) by capillary blotting. PCR probe labeling with DIG-11dUTP, hybridization, and NBT/BCIP chromogenic signal detection were carried out according to the manufacturer's instructions (DIG Northern starter kit; Roche Diagnostics, Mannheim, Germany). The labeled probe was synthesized with the primers complementary to the genomic region between FLJ37266 and DLG2 genes (primers intergene_spec-F and intergene_spec-R in Table 1). DIG-labeled RNA Molecular Weight Marker II 1.6–6.95 kb (Roche Diagnostics) was used to estimate the size of the bands by logarithmic relationship between molecular weight and distance migrated.

## Results

### Differential expression of FLJ37266 and DLG2 genes in RO

**Figure 1:**
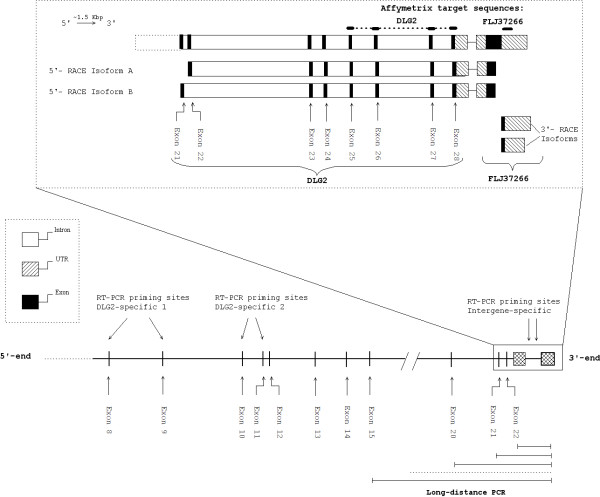
Schematic alignment of DLG2 gene isoforms, their genomic position, and localization of RT-PCR primers.

**Figure 2:**
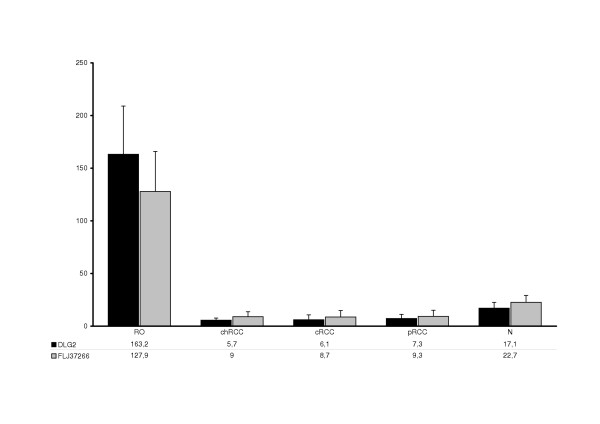
**Quantitative RT-PCR of Affymetrix target sequences for FLJ37266 (228973_at) and DLG2 (206253_at) genes in renal tumours**. The diagram shows the averaged level of expression as detected in 8 individual ROs, chRCCs, cRCCs, pRCCs and normal renal tissues (N). Both genes are significantly upregulated in ROs in comparison to other types of tumours and normal kidney samples (p < 0.05, Student's t-test).

The expression profiling with Affymetrix GeneChip array revealed a high number of genes expressed in both ROs and chRCCs (data not shown), but only few genes expressed exclusively in RO. Among them, FLJ37266 and DLG2 were most strikingly up-regulated in RO (Log2 ratios 8.1 and 7.8 in comparison to normal kidney, respectively). Both genes are represented by single target sequences in Affymetrix HG U133 set: FLJ37266 target sequence (probe ID: 228973_at) is located at 3'-UTR of FLJ37266 gene, and the target sequence for DLG2 gene (probe ID: 206253_at) covers four 3'-terminal exons of this gene (Fig. 1). Semiquantitative RT-PCR of FLJ37266 and DLG2 gene with the primers complementary to the target sequences (Affy_DLG2 and Affy_FLJ37266 primer pairs, Table 1) confirmed the results of microarray hybridization (Fig. 2).

### Single mRNA transcript of FLJ37266 and DLG2 genes

According to Ensembl genome database, DLG2 and FLJ37266 genes are located at the "minus" strand of chromosome 11q14.1 region only less than 1.5 kb to each other. The PCR was performed from RO cDNA as a template with forward primer specific to DLG2 gene and reverse primer specific to FLJ37266 gene (Affy_DLG2-F and Affy_FLJ37266-R, Table 1.) in one reaction. The amplification product of ~4.7 Kb suggests a single mRNA transcript for FLJ37266 and DLG2 genes.

Rapid amplification of cDNA ends was performed for FLJ37266 gene. After cloning and sequencing of the RACE PCR products, two variants of 5'-end and two variants of 3'-end of FLJ37266 were found. The sequences are deposited at dbEST database under the Accession Nrs. CX734843-CX734846. The alignment of the RACE sequences with genomic DNA and known mRNA sequences was performed using the BLAT tool [17]. The schematic representation of the alignment is given at Figure 1. The 3'-RACE variants of FLJ37266 gene are differed only with the length of the 3'-UTR whereas the 5'-RACE isoforms are differed by the termination at the exon 21 or 22 at the 5'-end. These alternative exons are present in homologous DLG2 mRNAs as well. The most prominent feature of 5'-RACE isoforms is that they contain the sequence complementary to the genomic DNA between the FLJ37266 and DLG2 genes. This implies that, the spliced mRNA transcript combining the whole FLJ37266 gene, intragenic region, and 3'-part of DLG2 gene is expressed in RO.

**Figure 3:**
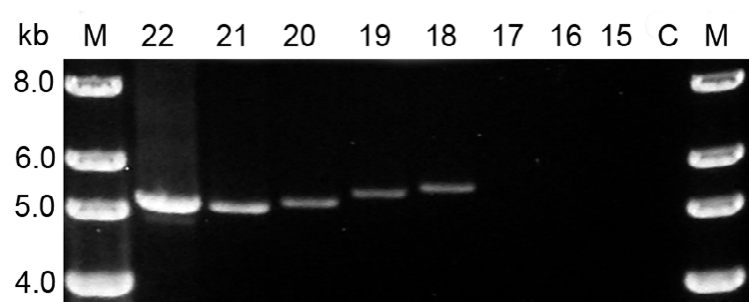
**Exon-specific LD PCR of the new DLG2 gene isoform**. M – molecular marker; exon-specific PCR products for the exons 22–15 as it was shown in Fig. 1; C – negative control.

The expression level of the 5'- RACE isoforms was studied by long-distance (LD) RT-PCR with the forward primers specific to the DLG2 exons 15–22 and the reverse primer specific to the FLJ37266 gene (LD primers, Table 1). LD RT-PCR (Fig. 3) showed that the most abundant variant of 5'-end of FLJ37266-DLG2 transcript corresponds to the RACE isoform A as it was shown in Figure 1. As revealed by LD PCR, besides the two 5'-RACE isoforms, some longer variants of FLJ37266-DLG2 transcript that contain additional exons at the 5'-end of DLG2 gene are also expressed at low level. Probably, due to their low abundance they were not detected among the cloned RACE products. In case of the 3'-RACE, two isoforms were amplified with quite similar efficiency, which points to their equal abundance.

**Figure 4:**
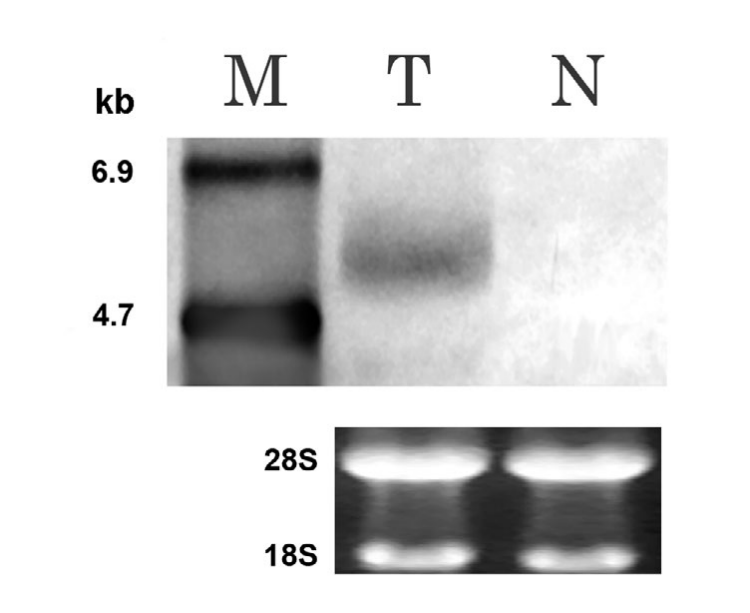
**Northern blot hybridization specific to the new DLG2 isoform**. M – RNA molecular weight marker; T – RNA sample extracted from a renal oncocytoma; N – RNA sample from the corresponding normal adult kidney.

The size of FLJ37266-DLG2 transcript was also estimated by Northern blot hybridization (Fig. 4). The calculated length of the transcript varies from 5.4 to 5.7 Kb, which roughly corresponds to the cumulative size of 3'- and 5'-RACE sequences. The part of the FLJ37266 -DLG2 transcript, containing the whole FLJ37266 gene, intergene region, 3'-UTR and two 3'-terminal exons of DLG2 gene was amplified using the primers Amp_F and Amp_R (Table 1) from three RO and counterpart normal kidney cDNAs, then cloned, and sequenced (dbEST Accession Nrs.: CX727314-CX727319). The accurate examination of the amplicon sequence revealed no nucleotide differences in comparison to cDNA sequences of FLJ37266 and DLG2 genes. The part of the amplicon, corresponding to the intergene region is completely identical to the genomic DNA, and has multiple stop codons. Thus, the FLJ37266-DLG2 mRNA transcript does not contain uninterrupted ORF. The longest ORF was found at the 5'-part of the transcript and corresponds to the coding region of DLG2 gene.

### Differential expression of FLJ37266-DLG2 transcript and DLG2 gene

**Figure 5:**
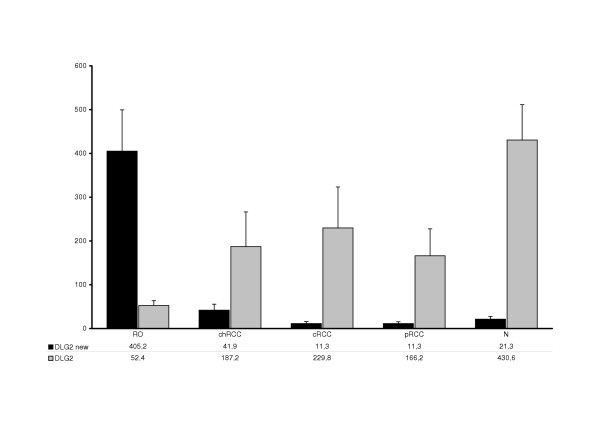
**Quantitative RT-PCR of the novel and known isoforms of DLG2 gene in renal tumours**. The diagramms show the averaged level of expression of the two DLG2 isoforms in 8 individual ROs, chRCCs, cRCCs, pRCCs and normal renal tissues (N). The new isoform of DLG2 is significantly upregulated in ROs in comparison to normal kidney and other types of tumours (p < 0.001, Student's test). The known isoform of DLG2 was significantly downregulated in ROs in comparison to normal kidney samples (p < 0.005, Student's test) but not against other types of tumours.

Microarray hybridization and RT-PCR with the primers specific to the Affymetrix target sequences corroborated that FLJ37266 and DLG2 genes are both highly up-regulated in RO. However, the discrimination of the new and the known DLG2 isoforms was impossible because they share the same Affymetrix DLG2 target sequence. Therefore, isoform-specific primers were designed. The primers specific to the new FLJ37266-DLG2 transcript were complementary to the intergene region (primers Intergene_spec-F and -R, Table 1). Two primer pairs specific to the known DLG2 isoform were designed for the exons present only in this isoform (exons 8 and 9 – DLG2_spec1 primers and exons 10 and 11 – DLG2_spec2 primers, Table 1). As shown in Figure 5, the opposite pattern of expression change in RO was observed for the different DLG2 isoforms. FLJ37266-DLG2 transcript is significantly up-regulated in RO in comparison to the normal kidney and other tumours. The conventional isoform of DLG2 gene is down-regulated in RO only, whereas the difference between the normal kidney and the carcinomas is statistically non-significant.

## Discussion

Although the global gene expression of over 200 renal cell tumours has already been studied, only 6 renal oncocytomas were included in these series [18-20]. The array analyses revealed expression profiles in distinct types of tumours, such as cRCCs, pRCCs, chRCCs but no specific expression for RO has been reported. We found that the whole FLJ37266 gene together with the 3'-end of DLG2 gene and the genomic region between them are transcribed as a single spliced mRNA in renal tissues. During preparation of the manuscript, mRNA sequence CR933674 with similar structure was announced at GeneBank database. We suggest that FLJ37266 gene is a part of a new isoform of DLG2 gene rather than a separate gene.

DLG2 belongs to a family of membrane associated guanylate kinases (MAGUKs). MAGUKs are thought to be scaffolding molecules playing the central role in establishing and maintaining the cell polarity in both epithelial cells and neurons. DLG2 shares the typical structure of MAGUK proteins including guanilate kinase (GUK), Src homology 3 (SH3), and three PDZ domains [21,22]. The longest ORF found in the new DLG2 isoform encodes GUK and SH3 domains, but not the PDZ domains located at the N-terminus.

The up-regulation of the novel DLG2 isoform was confirmed by different methods including microarray hybridization, RT-PCR, and Northern blot hybridization. The specificity of differential expression allows to consider this DLG2 isoform as a potential molecular marker for the diagnosis of RO. Especially valuable point is the very low expression of the new isoform not only in normal kidney, but also in other types of renal tumour, including chRCC. On the other hand, the results of RT-PCR indicate the expression of the known isoform of DLG2 in all other types of renal cell cancer whereas RO shows a low expression level.

## Conclusion

Although the functional role of the new DLG2 isoform in ROs is not yet known, it may be a good candidate for molecular differential diagnosis of RO. It is highly expressed in RO and show a low expression level in other types of renal cell tumours and normal kidney tissues whereas the DLG2 shows an opposite expression pattern. The significance of the differential expression in the routine pathology will be improved after arising isoform specific antibodies and immunohistochemical analysis of a large series of tumours.

## Competing interests

The author(s) declare that they have no competing interests.

## Authors' contributions

DZ carried out the gene cloning and expression studies and wrote the MS, ZS prepared the RNA and cDNA and sequenced the PCR products, GK selected the tissues and genes for analysis and coordinated the study.

## Pre-publication history

The pre-publication history for this paper can be accessed here:



## Figures and Tables

**Table 1:** Sequences of oligonucleotide primers

**PCR and sequencing primers**		
	**Name**	**Sequence 5'->3'**

RT-PCR	DLG2_spec1-F	GATGACCCTGGCATATTTATTACGA
	DLG2_spec1-R	ACGATAGACCCTGCTTCCTTCA
	DLG2_spec2-F	TGGGGAACCAACACATTCCT
	DLG2_spec2-R	TTGCTACTGCCTCTTCGTGTG
	Intergene_spec-F	GGCCAAAATGCTAAATGGTCCT
	Intergene_spec-R	CCTCACTTACCTCCCTTCCACA
	Affy_DLG2-F	GCTCTATCCCATTGCCATCTT
	Affy_DLG2-R	CGTTGTCAGAGGCGCAGTAT
	Affy_FLJ37266-F	TGCCAGATCGTGTAACTGGTG
	Affy_FLJ37266-R	TAGCCAAGAAATCCCGCAAA
		
RT-PCR controls	GAPDH-F	GGAAGGTGAAGGTCGGAGTCAAC
	GAPDH-R	CCACTTGATTTTGGAGGGATCTCG
	β-actin-F	ATGGATGATGATATCGCCGCGC
	β-actin-R	TGATGGTGGGCATGGGTCAGAA
		
Sequencing	Amp_F	CGTCTCTAACAGAGGAACAAGCCAAG
	Amp_R	GGAGTGTAAGCTGTGCAGTGTCTC
	DLG2_3'-F_2_ IRD800	GAAGCCATGAAGGAAACAGAGG
	DLG2_3'-F_IRD700	TTGATTGCCTATTTTGCCATTT
	DLG2_5'-F_ IRD700	ACCAGCTTTCGCTATGCAAC
	FLJ37266-F_IRD800	TGTTGATGTCTCTATGGCTCCA
	FLJ37266-R_IRD700	TGGAGCCATAGAGACATCAACA
		
LD-PCR	FLJ37266_universal-R	CAATTCTGTGGGAGGAGAGCAA
	DLG_exon22-F	GGTGACGACGGTTATGGAACAA
	DLG_exon21-F	ACAAGGAGCAGAGTGAGCAGGA
	DLG_exon20-F	GTGCCCGATTGAAGACAGTGAA
	DLG_exon19-F	ACGACAAGAGCAAGGACAGTGG
	DLG_exon18-F	CCATGACCTACGAGAGCAGATGA
	DLG_exon17-F	GGCTGGACAGACAGTGACGATT
	DLG_exon16-F	GTGTCCTTCATTCTGGCTGGTG
	DLG_exon15-F	ACATAGCACCGCAACTCGTCAG
